# Innate Lymphoid Cells and Myocardial Infarction

**DOI:** 10.3389/fimmu.2021.758272

**Published:** 2021-11-11

**Authors:** Wenling Yang, Jibin Lin, Jin Zhou, Yuqi Zheng, Shijiu Jiang, Shaolin He, Dazhu Li

**Affiliations:** Department of Cardiology, Union Hospital, Tongji Medical College, Huazhong University of Science and Technology, Wuhan, China

**Keywords:** myocardial infarction, innate lymphoid cells, myocardial ischemia-reperfusion injury, regeneration and repair after myocardial infarction, the acute phase of myocardial infarction

## Abstract

Myocardial infarction results from obstruction of a coronary artery that causes insufficient blood supply to the myocardium and leads to ischemic necrosis. It is one of the most common diseases threatening human health and is characterized by high morbidity and mortality. Atherosclerosis is the pathological basis of myocardial infarction, and its pathogenesis has not been fully elucidated. Innate lymphoid cells (ILCs) are an important part of the human immune system and participate in many processes, including inflammation, metabolism and tissue remodeling, and play an important role in atherosclerosis. However, their specific roles in myocardial infarction are unclear. This review describes the current understanding of the relationship between innate lymphoid cells and myocardial infarction during the acute phase of myocardial infarction, myocardial ischemia-reperfusion injury, and heart repair and regeneration following myocardial infarction. We suggest that this review may provide new potential intervention targets and ideas for treatment and prevention of myocardial infarction.

## Introduction

Myocardial infarction (MI) is one of the main causes of death worldwide, is characterized by high morbidity and mortality, and poses a serious threat to human health (1). The pathophysiological basis of MI is atherosclerosis (AS), which is a chronic inflammatory disease characterized by formation of fibrofatty lesions (2). The ‘Canakinumab Anti-inflammatory Thrombosis Outcomes Study’ (CANTOS) demonstrated the clinical benefits of suppressing inflammation (3). In the immune microenvironment of AS, interactions between innate immune, adaptive immune, and non-immune cells promote progression of AS (4). Innate lymphoid cells (ILCs) are a family of immune cells recently shown to be implicated in inflammation, metabolism and tissue remodeling (5), and cardiovascular diseases such as AS (6–8), pericarditis (9), cardiac fibrosis (10)and MI (11). Gong et al. summarized the roles of helper ILCs in inflammation-associated cardiovascular disease (12). However, the specific roles of ILCs in MI are still unclear. To identify potential intervention targets and propose ideas for treatment and prevention of MI, this review explores the relationship between ILCs and MI during the acute phase of MI, myocardial ischemia-reperfusion injury, and heart repair and regeneration following MI.

## Types of ILCs

ILCs are mainly distributed in the intestines, lungs and other mucosal layers that are in direct contact with the environment, and participate in the immune system’s first line of defense against pathogens. ILCs lack cell surface molecules that recognize other lymphocytes and cannot recognize antigens. They produce cytokines such as interferon-γ (IFN-γ), interleukin (IL)-4, IL-5, and IL-13, which play an important role in tissue homeostasis and inflammation (5). The types and functions of ILCs are shown in **Table 1**. Depending on their killing ability, ILCs can be divided into cytotoxic ILCs, namely conventional natural killer (NK) cells, and auxiliary ILCs such as type 1 (ILC1), 2 (ILC2), and 3 (ILC3) ILCs. Based on differences in expression of key transcription factors and cytokines, ILCs are divided into three subgroups: Groups 1, 2, and 3. Group 1 ILCs (including ILC1s and NK cells) are functionally similar, express the T-box transcription factor T-bet, produce IFN-γ and tumor necrosis factor (TNF), and react to tumors and intracellular pathogens such as viruses. Group 2 ILCs include cytokine-producing T_h_2 cells, whose development and function depend on GATA binding protein 3 (GATA3) and retinoic acid receptor-related orphan receptor-α (RORα), and ILC2s, which mainly act on extracellular parasites and allergens and participate in repair of tissue and organ damage. Group 3 ILCs include ILC3s and lymphoid tissue inducer (LTi) cells, which produce IL-22 and IL-17 cytokines to resist bacteria, fungi and other extracellular microorganisms (13).

**Table 1:** Types and functions of the ILCs.

Type	Group 1 ILC		Group 2 ILC	Group 3 ILC		ILCreg
	NK Cell	ILC1s	ILC2s	ILC3s	LTi	
**Transcription factors**	T-bet		RORα and GATA3	RORγt		Id3 and Sox4
**Secretion**	IFN-γ and TNF		IL-4, IL-5 and IL-13	IL-17A and IL-22		IL-10 and TGF-β1
**Function**	Anti-tumor and anti-viral functions, immune regulation, hypersensitivity, autoimmune diseases, etc.	Defense against parasites and intracellular bacteria, killing tumor cells.	Respond to extracellular parasites and allergens; participating in repair of tissue and organ damage, treatment of respiratory diseases, allergic inflammation, etc.	Defense against bacteria, fungi and other extracellular microorganisms, maintain intestinal stability.		Regulate congenital intestinal inflammation.

Recently, ILCregs were identified as belonging to a new distinct regulatory subset of ILCs. ILCregs suppress activation of ILC1s and ILC3s *via* secretion of IL-10 and are inhibitory during innate intestinal inflammation (14). ILCregs can also promote colorectal tumor growth from ILC3 transdifferentiation. Blockade of TGF-β signaling disrupts conversion of ILCregs and inhibition of tumor growth (15). The roles of NK cells in MI have been reviewed elsewhere (16) and therefore are not discussed here.

## The Distribution of ILCs in the Heart and Surrounding Tissues

Cardiac ILCs are a type of static and undifferentiated tissue-resident cell that lack a clear immunophenotype, have unique surface markers and self-renew quickly when disturbed (17). Most research on the tissue distribution of ILCs has focused on ILC2s. Perry et al. (18) reported that NH cells (one type of ILC2) were present in aortic perivascular adipose tissue. These adipose tissue-resident NH cells could be stimulated by IL-33 to produce IL-5. Engelbertsen et al. (19) demonstrated that ILC2s were present in the aorta and in perivascular adipose tissue. Newland et al. (7) showed that ILC2s were present in para-aortic adipose tissue and lymph nodes. Wu et al. reported auxiliary ILCs (ILC1s, ILC2s and ILC3s) in the aorta in response to a high fat diet (10). Gao et al. (6), Deng et al. (17) and Chen et al. (10) reported expression of ILC2s in the heart. Bracamonte-Baran et al. (20) found that in the hearts of healthy humans and mice, non-cytotoxic ILCs were predominantly a type 2-committed population with progenitor-like features and could differentiate into conventional ILC2s during myocarditis and ischemia.

## The Role of ILCs in Various Stages of MI

It is known that the immune system is activated during myocardial injury. In the coronary arteries, exposed atherosclerotic plaques promote recruitment and entry of cells of the innate immune system into heart tissue, prompting release of cytokines and aseptic inflammation in damaged heart tissue (21). Immune cells participate in the cleanup of necrotic debris, and at the same time initiate a repair response in the myocardium. However, excessive activation of immune cells in heart tissue may lead to cardiomyocyte apoptosis and fibrosis (22). In this section, we summarize the roles ILCs play in the acute phase of MI, myocardial ischemia-reperfusion injury, and repair and regeneration of heart tissue after MI.

## The Role of ILCs in AS

As is well known, AS is the pathologic basis for MI. Several studies have reported roles for ILCs in AS. For ILC1s, Wu et al. (10) showed that ILC1s can aggravate AS. When depleted of ILC1 cells, the atherosclerotic lesions shrank in Apoe^-/-^Rag1^-/-^ mice, and this effect could be rescued through adoptive transfer of ILC1s. In the case of ILC2s, IL-5 and IL-13 are cytokines that play a central role in their function. Perry et al. (18) demonstrated a role for the ILC2-IL-5-Bl-IgM axis in AS. IL-33 stimulated NH cells to secrete IL-5; IL-5 then stimulated B-1a B cell proliferation to produce IgM natural antibody, which is an atheroprotective factor. Engelbertsen et al. (19) demonstrated that expansion of CD-25-expressing ILCs could reduce AS, and was associated with reduced VLDL and increased IL-5. Global depletion of ILCs did not affect lesion size, indicating that different subsets of ILCs may have different roles in AS, consistent with the report of Wu (10). Newland et al. (7) showed that genetic ablation of ILC2 could accelerate development of AS, which could be prevented by reconstitution with ILC2 (wild type) but not IL5^-/-^ or IL13^-/-^. They found that ILC2-derived IL-5 and IL-13, and especially IL-13, were critical for control of AS progression, partly through M2 polarization. Therefore, the ILC2s-IL-13-M2 axis could be another pathway for ameliorating AS. Gao et al. (6) reported that Treg-ILC2s-IL13 could improve AS. Tregs may play a partially protective role against AS by expanding the number of ILC2s and thereby increasing IL-13 production. We are not aware of any studies on ILC3s and ILCregs in AS. ILC3s can secrete IL-17 and IL-22, but reports of the roles of these two cytokines in AS are inconsistent (23). In addition, given the small numbers of ILC3s, their role in AS is unclear. ILCregs can produce IL-10 and TGF-β, which are protective factors for AS. Therefore, ILCregs may have the potential to improve AS, but further studies are needed.

## The Role of ILCs in the Acute Phase of MI

During acute MI, coronary blood flow is suddenly blocked and the myocardium goes from ischemic to necrotic. Although several types of immune cells are involved in this process, the roles and mobilization of ILCs as “front-line” innate immune cells in acute MI are not clear.

Recently, Li et al. (24) described levels of ILCs in the peripheral blood in the setting of acute ST-segment elevation myocardial infarction (STEMI). They consecutively enrolled 176 STEMI patients and 52 control patients, initially collecting blood samples after the diagnosis and prior to medical therapy, and following with serial samples at days 3, 5 and 14 after the onset of STEMI. During this period, the proportion of total ILCs and ILC1s increased compared with controls; while the proportion of ILC2s decreased significantly. In addition, patients were followed for up to 23 months. They found that ILC1s were an independent predictor of major adverse cardiovascular events. In addition, RNA sequencing performed on ILC1s showed that IFN-γ, TNF-α, vascular cell adhesion molecule 1 (VCAM1), and matrix metallopeptidase 9 also increased. These results suggested the possibility of using ILCs as a disease marker in the future.

Several papers have focused on ILC2s in AS (7, 10, 11), but few in MI. Bracamonte-Baran (20) reported the presence of ILC2s during the early stages of ischemic injury. During ischemia, cardiac ILCs could differentiate into ILC2s, but not ILC1s or ILC3s. Importantly, they demonstrated that the ILC2s resulted from local proliferation rather than infiltration of circulating ILCs. Yu et al. (11) demonstrated that low-dose IL-2 injection could activate ILC2s in acute coronary syndrome (ACS). Individuals received low or medium-dose IL-2 or placebo daily for 5 days. The placebo had no effect on ILC2s, meanwhile, IL-2 injection could decrease ILC2 canonical surface markers among isolated peripheral blood mononuclear cells (PBMCs) and increase serum IL-5 titers, indicating activation of ILC2s. IL-5 plays an anti-atherosclerotic effect by increasing the titer of a natural IgM antibody specific to the oxidized LDL epitope (25). IL-13, another cytokine secreted by ILC2s, was reported to be significantly increased in the myocardium after MI, with a peak on day 3 (26). IL-13 could improve cardiac function by recruiting more monocytes/macrophages and inducing M2 macrophages (27). IL-13 could also reduce cardiac scar area (28), increase cardiomyocyte cell cycle activity (28) and even facilitate cardiac regeneration (29). It has been reported that IL-13 may also be a prognostic marker of acute myocardial infarction (AMI) (30). These results indicate that ILC2s may play vital roles in the acute phase of MI.

There are as yet no reports of a role for ILC3s in MI. However, the relationship between ILC3-secreted IL-17 and MI has been well documented (31). What’s more, expression of another ILC3-produced cytokine, IL-22, was elevated in ACS (23). Tang et al. (32) reported that IL-22 could prevent LV dysfunction and heart failure after acute MI. The question of whether ILC3s have any effect on MI requires further research. The same question arises for ILCregs in terms of the known roles of IL-10 and TGF-β in MI (14).

## The Role of ILCs in Myocardial Ischemia-Reperfusion Injury

Despite the increasing use of coronary reperfusion in interventional therapy, morbidity and mortality after STEMI remain high. The mechanisms of myocardial ischemia-reperfusion injury (MIRI) are unclear. Apart from cardiomyocyte cell death, coronary microvascular injury is also an important component of MIRI (33, 34). Manifestations of coronary microvascular injury during MIRI range from reversible edema to capillary destruction with intramyocardial hemorrhage. Cardioprotection refers to all measures and interventions to reduce myocardial ischemia and reperfusion injury. Ischemic conditioning, including remote ischemic conditioning, was reported to reduce inflammation, and was considered to be one of the main methods of cardioprotection (34, 35). There is currently no research directly connecting ILCs to MIRI, but there are some reports characterizing ILCs in ischemia-reperfusion injury to other organs. Kang et al. (36) reported that ILC1s increased in high-fat diet mice following liver ischemia-reperfusion injury (IRI), with an increased production of IFN-γ and TNF-α. Cao et al. (37) reported that ILC2s could prevent renal injury in mice subjected to IRI, and that this effect was associated with M2 macrophage induction and ILC2 production of amphiregulin. Subsequently, the same group showed that ILCregs could suppress innate immunity and reduce renal IRI (38). IL-2/IL-2 antibody complexes (IL-2C) could promote ILCreg expansion *in vivo* and prevent renal IRI in Rag^-/-^ mice. Using anti-CD25 antibody to deplete ILCregs abolished these beneficial renal effects. Adoptive transfer of ILCregs improved renal function. For ILC3s, Eggenhofer et al. (39) reported that NCR^+^ ILC3s could protect against hepatic IRI through IL-22. Severe hepatic IRI in NCR^+^ ILC3-deficient mice can be reversed with adoptive transfer of IL-22-producing NCR^+^ ILC3s. What’s more, Geha et al. (40) reported that ILC derived IL-17A was essential for intestinal IRI.

Studies in a mouse model of MIRI demonstrated that the inflammatory cytokines TNF-α, IL-1β and IL-6 were rapidly up-regulated after reperfusion, but then decreased 6 to 24 hours later. 24–72 hours after MIRI, expression of pro-inflammatory cytokines decreased significantly, and levels of fibrotic mediators such as TGF-β and IL-10 showed a continuous and rapid increase, reflecting suppression of acute inflammation and transition to cardiac repair and proliferation (41, 42). Recently, Pluijmert et al. (43) summarized the multifactorial and dynamic process of the inflammatory response in MIRI, including TNF-α, IL-4, IL-13, IL-10 and TGF-β. These results suggested that cytokines, especially those related to ILCs, play important roles in MIRI (22).

IL-33 is a member of the IL-1 family that plays an important role in innate immunity. The IL-33/ST2 signaling pathway mainly acts to reduce cardiac hypertrophy, ventricular dilatation and cardiac fibrosis under mechanical stress. IL-33 can stimulate secretion of IL-5, IL-13, BMP-7, IL-10 and G-CSF in ILC2s through the ST2 receptor, and has a protective effect on heart tissue damage. In acute heart injury, IL-13 and BMP-7 promote an IL-33-mediated anti-fibrosis response, immune cell recruitment and tissue repair (10). Studies have shown that circulating levels of IL-33 and soluble ST2 (sST2) are related to the severity of ACS. The level of IL-33 in serum is significantly lower in patients with ACS compared to those with stable angina pectoris. The level of sST2 is negatively correlated with left ventricular ejection fraction and prognosis of MI patients, and reflects the severity of MI (44). In a rat MIRI model, subcutaneous injection of IL-33 can significantly reduce infarct size and myocardial fibrosis. IL-33 exerts a cardioprotective effect by binding to the ST2 receptor (45). The IL-33/ST2 system is closely associated with ILC2s, and may be a potential target for predicting severity and prognosis of ACS and treatment of MIRI (44). Koeppen et al. (46) demonstrated that amphiregulin can suppress MIRI. It has also been shown that ILC2s can produce amphiregulin (37, 47). The IL-33-ILC2-AREG pathway could protect against intestinal inflammatory diseases (48). So, it is possible that the ILC2s-AREG pathway is involved in MIRI, although further research is needed. Liao et al. (49) demonstrated that IL-17A could promote cardiomyocyte apoptosis and neutrophil infiltration after MIRI *in vivo*. IL-17A knockout or anti-IL-17A monoclonal antibody treatment may significantly improve MIRI *via* reduced infarct size, reduced cardiac troponin T levels, and improved cardiac function. Whether ILC3s can affect MIRI through IL-17A is unknown.

In short, although critical evidence is lacking for an important role of ILCs in MIRI, ILC2s may be the most promising candidate.

## The Role of ILCs in Repair and Regeneration After MI

The hearts of adult mammals cannot regenerate. After MI, necrotic areas gradually become fibrotic to form non-contractile scars, leading to heart failure and even death (50). Innate immune cells (such as macrophages, mast cells and ILCs) curb inflammation and regulate the balance between cardiac tissue repair and regeneration and scar formation by removing dying cells and promoting cardiomyocyte replacement (51). After myocardial ischemia injury, innate immune cells residing in the myocardium are immediately activated. Resident cardiac macrophages have been shown to initiate apoptosis after 2 hours, and circulating monocytes to infiltrate into the injury site and differentiate into macrophages (52). M1 macrophages are responsible for degradation of the extracellular matrix and removal of cell debris. M2 macrophages secrete anti-inflammatory cytokines and promote angiogenesis and collagen deposition (51). Mast cells release histamine to trigger vascular permeability and migration of circulating innate immune cells.

**Figure 1:**
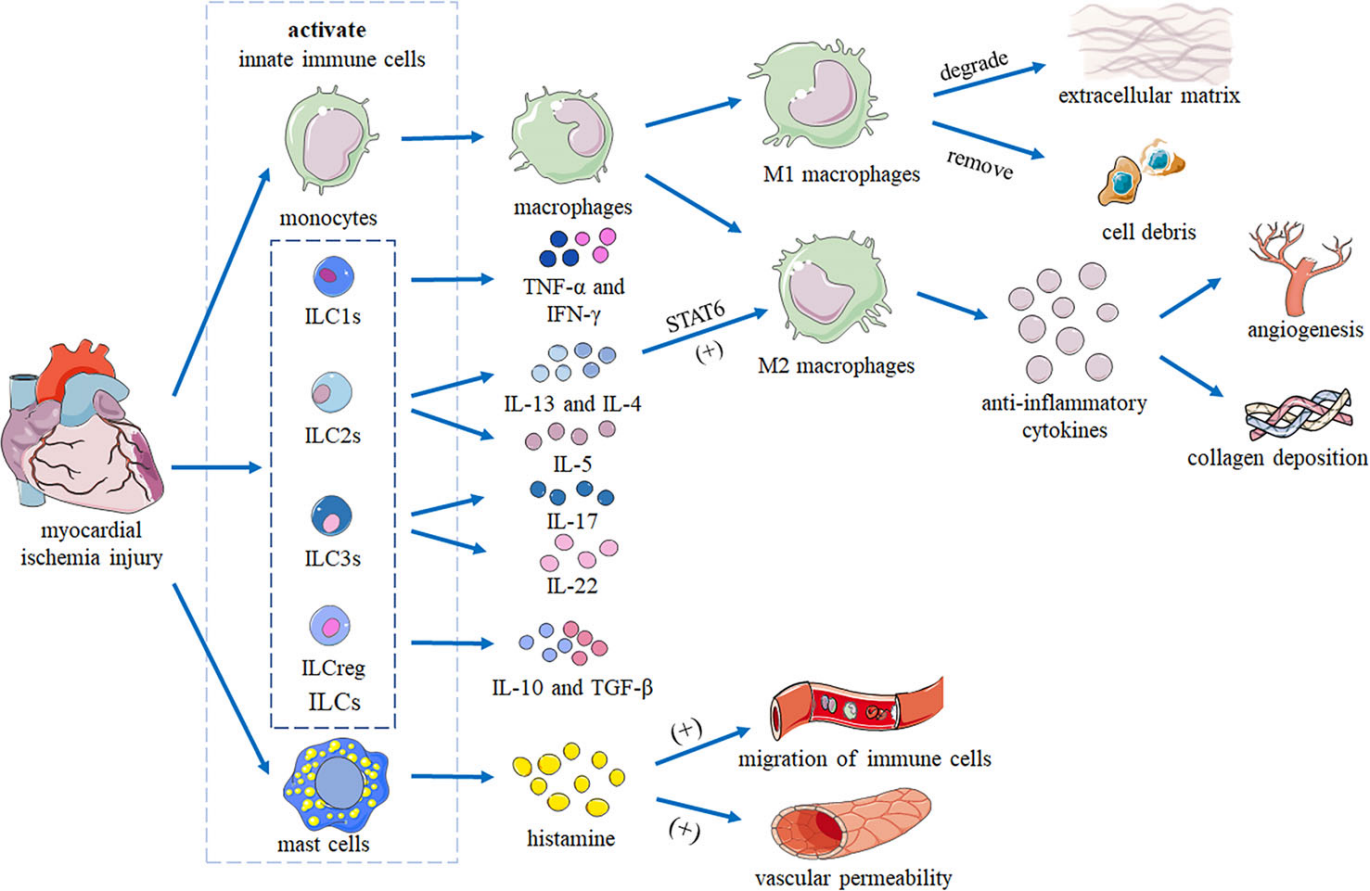
Heart repair by innate immune cells following myocardial ischemia injury. After myocardial ischemic injury, innate immune cells residing in the myocardium are immediately activated. Monocytes infiltrate the injury site and differentiate into macrophages. M1 macrophages are responsible for degrading the extracellular matrix and removing cell debris. M2 macrophages secrete anti-inflammatory cytokines to promote angiogenesis and collagen deposition. IL-13 and IL-4 produced by ILCs promote polarization of macrophages into the M2 phenotype by activating STAT6. Mast cells release histamine to trigger vascular permeability and migration of innate immune cells. Together, these cells coordinate removal of damaged cells, remodeling of the tissue matrix, and recruitment of additional immune cells from the blood to promote recovery of cardiac function.

ILCs and macrophages are both resident-tissue cells. ILCs can interact with macrophages through different signaling pathways. ILC2s can secret IL-4 and IL-13, which promote polarization of macrophages into the M2 phenotype through STAT6 by inducing phosphorylation and promoting transcription (53). This anti-inflammation macrophage phenotype has important roles in wound repair and resolution (54), which is also important for myocardial injury. Hams et al. (55) summarized the roles of ILCs in fibrosis with potential interactions between ILCs and macrophages. Kim (56) also discussed how ILCs coordinate the polarization of lung macrophages through cytokine secretion and ILC-macrophage interactions. So, it’s possible that ILCs can communicate with macrophages *via* IL-4/IL-13 secretion and direct cell–cell contact. Together, they coordinate the removal of damaged cells, the remodeling of the tissue matrix, and the recruitment of additional immune cells from the blood to promote recovery of heart function after MI (**Figure 1**) (51).

Recently, Yu et al. (11) reported that ILC2s could promote cardiac healing and recovery of ventricular function after MI. They found that ILC2 levels increased after MI in an ST2-dependent manner. In addition, they suggested that ILC2s can shift scar formation toward less intrusive remodeling in the early recovery period. In terms of mechanism, they found that the IL-2 axis was a major upstream regulator of ILC2s and was also central to ILC2 function following MI.

Cardiac fibrosis is an important process following MI. Chen et al. (10) reported that IL-33 treatment expanded cardiac ILC2s and elicited protective effects against catecholamine-induced cardiac fibrosis with reduced cardiomyocyte death, immune cell infiltration, tissue fibrosis, and improved myocardial function.

For the ILC2-related cytokine IL-5, Xu et al. (57) reported that it facilitates recovery of cardiac function post-MI by promoting eosinophil accumulation and subsequent CD206^+^ macrophage polarization *via* the IL-4/STAT6 axis. The ILC2-related cytokine IL-13 could regulate leukocyte recruitment and induce differentiation of M2-like monocytes/macrophages, and could promote recovery of cardiac function after MI by secreting anti-inflammatory cytokines to stimulate new blood vessel formation and collagen deposition, thereby enhancing wound healing in the infarct area (26).

In brief, the function of ILC2s in the post-MI period is becoming clear. More evidence is needed to address the roles of other ILCs following MI.

## Discussion and Prospects

In this review, we have summarized the complex relationships between ILCs and MI, and discussed evidence for their involvement in the acute phase of MI, MIRI, and repair and regeneration after MI. We also briefly described the spatiotemporal distribution of ILCs in MI. We suggested possible mechanisms by which ILCs play a role in MI by summarizing ILC-related cytokines and the involvement of ILCs in other similar diseases.

Functional analysis of the effects of different ILC subgroups found that ILC1s are involved in progression of AS, ILC2s exert an anti-atherosclerotic effect, while the effect of ILC3s on AS is still controversial (6–8). However, the roles and mechanisms of ILCs in MI have not been fully elucidated.

In patients with AMI (44), an increase in ILC1s that produce IFN-γ can be detected within 12 hours of the onset of symptoms, which is associated with a poor clinical prognosis. In the meantime, the proportion of ILC2s decreased significantly when compared to control group. ILC2s were also found to be involved in the early stages of ischemic injury (20). Yu et al. (9) demonstrated that low-dose IL-2 injection could activate ILC2s in ACS.

Despite the increasing use of coronary reperfusion in interventional therapy, morbidity and mortality after STEMI are still high. This may be related to damage to coronary microvascular cells during MIRI (33). Manifestations of coronary artery injury during MIRI range from reversible edema to capillary destruction with intramyocardial hemorrhage. Ischemic conditioning, which refers to short-term ischemia-reperfusion in the heart or other organs to reduce the area of MI and coronary microvascular damage (34), involves several immune factors and is one of the main methods of cardioprotection (35). Minimizing inflammation requires removal of cell debris and promotion of healing following MI, while unlimited inflammation impairs healing and induces adverse cardiac remodeling (58). In remote ischemic conditioning, the spleen is immediately activated *via* the vagus nerve, releasing myocardial protective factors and reducing infarct size (59). As we know, the spleen is one of the main sources of ILCs (60), so it is possible that splenic ILCs could play a role in remote ischemic conditioning.

Though not direct evidence for ILCs in MIRI, several groups (22, 41–43) have reported roles for ILCs in IRI and ILC-related cytokines in MIRI, suggesting that ILCs can play important roles in MIRI, with ILC2s being the most promising candidate.

The presence of ILCs in and surrounding the heart (18) enables these innate immune cells to function as “local fire captains” in cardiovascular diseases, consistent with their tissue-resident character. Changes in numbers of ILCs among PBMCs in STEMI patients, and their association with clinical prognosis, makes them a potential disease biomarker. However, Bracamonte-Baran et al. demonstrated that increases in ILC2 levels resulted from local proliferation rather than infiltration of circulating ILCs. The origin of ILCs requires further research.

It is known that cardiomyocytes are non-regenerative cells. So human embryonic stem cells and human induced pluripotent stem cells may have great potential in treatment of MI (61). Similarly, ILC progenitor cells can differentiate into different ILCs under certain conditions (62). They have been shown to differentiate into ILC2s but not ILC1s and ILC3s during ischemia (20). More research is needed into the role of ILC differentiation in MI.

Recently, Yu et al. (11) reported that ILC2s could promote cardiac healing and recovery of ventricular function after MI through the IL-2 axis. Chen et al. (10) reported that cardiac ILC2s provide protection from cardiac fibrosis and improve myocardial function. Therefore, ILC2s are involved in the entire process of MI, from AS and the early stage of cardiac injury to tissue remodeling and post-MI repair. More studies are however needed to better elucidate the roles of other ILCs in MI.

For future studies of ILCs, more suitable animal models such as tissue-specific gene knockout mice, and over-expression methods like accessible adoptive transfer systems, need to be established. In clinical research, not only patients with acute MI can be recruited, but also patients with stable angina, unstable angina and non-STEMI to further clarify the role of ILCs. In addition, in the era of single cell transcriptomics, big data analysis of ILCs should be explored. What’s more, communications between ILCs and other cells should be further investigated. Finally, the role of ILC plasticity in MI should be addressed, as should the role of ILCregs in MI.

In conclusion, ILC2s play important roles in MI, but more research is needed to further explore the mechanisms and roles of other ILCs in MI and to provide better treatment and prevention.

## Author Contributions

The paper presented was performed in collaboration with all authors. WY wrote the manuscript. JL edited the manuscript. JZ prepared the figure and table. YZ and SJ participated substantially in the organization and coordination of the paper. SH and DL devised the concept and supervised the whole study. All authors contributed to the article and approved the submitted version.

## Funding

This work was supported by the National Key R&D Program of China (no. 2017YFA0208000 to SH) and the National Natural Science Foundation of China (no. 82070317 to JL).

## Conflict of Interest

The authors declare that the research was conducted in the absence of any commercial or financial relationships that could be construed as a potential conflict of interest.

## Publisher’s Note

All claims expressed in this article are solely those of the authors and do not necessarily represent those of their affiliated organizations, or those of the publisher, the editors and the reviewers. Any product that may be evaluated in this article, or claim that may be made by its manufacturer, is not guaranteed or endorsed by the publisher.
